# Transient Global Amnesia Related to the Third Coronavirus Disease-19 (COVID-19) Vaccination

**DOI:** 10.7759/cureus.27121

**Published:** 2022-07-21

**Authors:** Masahito Katsuki, Yoichi Higo, Sakura Komagata, Kenta Kashiwagi, Akihito Koh

**Affiliations:** 1 Neurosurgery, Itoigawa General Hospital, Itoigawa, JPN; 2 Radiology, Itoigawa General Hospital, Itoigawa, JPN; 3 Neurology, Itoigawa General Hospital, Itoigawa, JPN

**Keywords:** mri images, comorbid anxiety, sars-cov-2 vaccination, covid-19 outbreak, transient global amnesia

## Abstract

We present a case report of transient global amnesia (TGA) after vaccinating the third dose of coronavirus disease-19 (COVID-19). A 65-year-old Japanese woman presented with TGA after her third vaccination, which spontaneously resolved. This article aims to facilitate the clinicians’ understanding that TGA could occur after the COVID-19 vaccination in the COVID-19 global outbreak.

## Introduction

Transient global amnesia (TGA) is a symptom of acute anterograde amnesia, which usually resolves within 24 h. Its incidence is estimated as 5-10 per 100,000 people per year. It often occurs under emotional or physical stress. The pathophysiology remains unclear: focal hippocampal ischemia, venous congestion, migraine- or epilepsy-like mechanisms, and metabolic stress have been hypothesized [1]. An acute increase in TGA cases was reported in Germany during the covid outbreak [2]. A similar trend was also reported in Japan [3]. TGA after nasopharyngeal swab for COVID-19 screening [4] and TGA as a first manifestation of COVID-19 were reported [5]. Two cases of TGA-presenting stroke after COVID-19 infection have been also reported [6]. However, TGA after COVID-19 vaccination has not been reported, so we herein describe such a case.

## Case presentation

**Figure 1:**
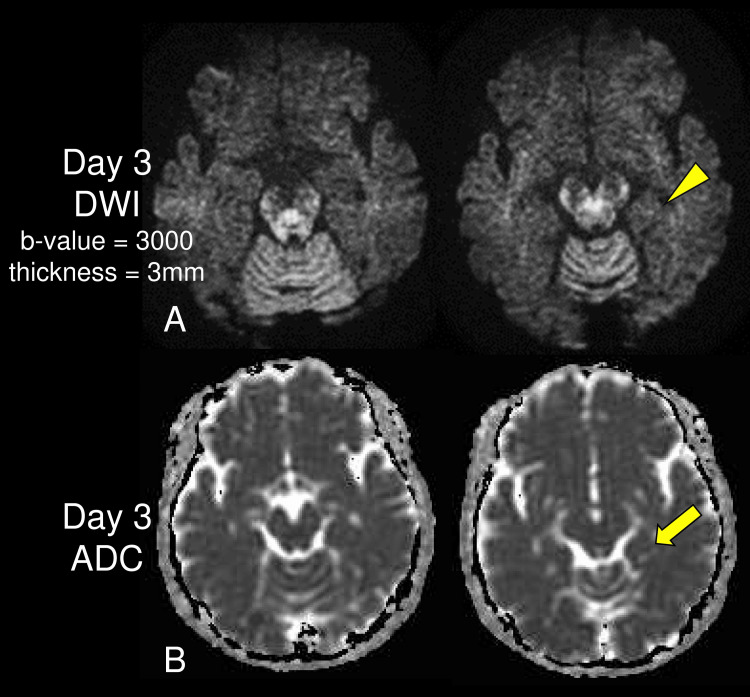
Magnetic resonance imaging on day 3 Diffusion-weighted images (DWI) on day 3 with a b-value of 3000 and thickness 3mm showed restricted diffusion in the left temporal lobes (arrowhead in A) along with corresponding apparent diffusion coefficient (ADC) images (arrow in B).

A 65-year-old Japanese woman with no previous medical history presented with confusion and anterograde amnesia 3 hours after her third COVID-19 vaccination [COVID-19 Vaccine Moderna Intramuscular Injection (Lot 000011A, Moderna, Takeda, and Moderna)]. She lived alone and was an elementary school teacher. She had not experienced any side effects of the vaccinations, but she felt undefinable anxiety during the third vaccination. Laboratory tests, head computed tomography, and magnetic resonance imaging (MRI) at the emergency room did not show any specific findings. She received previous 2-time vaccination [BNT162b2 (Lot FC5295, Comirnaty, BioNTech and Pfizer)] 8 months ago and [BNT162b2 (Lot FC9880)] 7 months ago. Her amnestic symptoms improved after 12 hours. She remembered that she had the third vaccination, but other memories during TGA were lost. Vitals signs were all within normal limits consistently. MRI on the third day was performed. Diffusion-weighted images on day 3 with a b-value of 3000 and thickness of 3mm showed restricted diffusion in the left temporal lobes (arrowhead in Figure 1A) along with corresponding apparent diffusion coefficient images (arrow in Figure 1B).

Magnetic resonance angiography did not reveal any atherosclerosis changes or abnormality. Electroencephalography, arterial spin labeling imaging, electrocardiograph, and echocardiography did not show any abnormal findings. No recurrence has been experienced for this one month.

## Discussion

This is the first report of TGA related to the third dose of COVID-19 vaccination. We believe that the third dose of the vaccine and its stress response may have acted as a TGA trigger.

The adverse events reported after vaccination include headache, fatigue, and nausea were common neurological symptoms occurring in over 50% of vaccinated individuals [7]. Vaccination is associated with pain, and emotional stress, directly related to discomfort. Also, worry about its adverse effects was strengthened in the pandemic context. Furthermore, the sensational daily news on social media about the adverse effects of vaccination and unfounded remarks on social networking services have promoted its anxiety. These stress-associated triggers, such as psychological and emotional stress, may lead to hippocampal dysfunction through several mechanisms involving glutamate neurotoxicity, the release of vasoactive stress hormones like angiotensin I and II, and oxidative stress [1].

As another mechanism, thrombosis caused by the COVID-19 vaccination may be hypothesized. Previously, cerebral vein thrombosis 7 to 10 days after vaccination was reported [8]. The patients with thrombosis had high antibodies to platelet factor 4-polyanion complexes, which led to thrombosis via platelet activation. Also, the vaccine uses adenovirus to transfer messenger ribonucleic acid. Adenovirus itself can combine with platelet, leading to thrombosis [9]. Also, viral spike glycoprotein binds to angiotensin-converting enzyme 2 (ACE2), resulting in ACE2 downregulation, which in turn overactivated the classical renin-angiotensin system (RAS) and activates the alternative RAS signal in the brain, resulting in a cascade of coagulopathy and inflammation [6]. The true mechanism is uncertain, but the vaccine’s coagulopathy seemed hypothesized. Apart from TGA, some population-based surveys at the COVID-19 vaccination venues have been also reported [10,11], so further research on the side effects of the COVID-19 vaccination like this and their follow-up should be reported.

## Conclusions

We experienced the case with TGA after the third COVID-19 vaccination. A 65-year-old Japanese woman showed TGA after her third vaccination, which spontaneously resolved. This article aims to facilitate the clinicians’ understanding that TGA could occur after vaccination in the COVID-19 global outbreak. We should be careful of TGA after vaccination. More reports are needed to investigate pathogenesis and neuroimaging findings in such TGA patients.
